# Equity in adherence to and effect of prenatal food and micronutrient supplementation on child mortality: results from the MINIMat randomized trial, Bangladesh

**DOI:** 10.1186/1471-2458-14-5

**Published:** 2014-01-07

**Authors:** Rubina Shaheen, Peter Kim Streatfield, Ruchira Tabassum Naved, Lars Lindholm, Lars Åke Persson

**Affiliations:** 1 International Maternal and Child Health, Department of Women’s Health, Uppsala University, Akademiska sjukhuset, SE 751 85 Uppsala, Sweden; 2 icddr,b: International Centre for Diarrheal Disease Research, Bangladesh, 68 Shaheed Tajuddin Ahmed Sarani, Mohakhali, Dhaka 1212, Bangladesh; 3 Department of Public Health and Clinical Medicine, Epidemiology and Global Health, Umeå University, Umeå, Sweden

**Keywords:** Equity, Child mortality, Food supplementation, Micronutrient supplementation, Adherence, Bangladesh

## Abstract

**Background:**

Evidence is often missing on social differentials in effects of nutrition interventions. We evaluated the adherence to and effect of prenatal food and micronutrient supplementations on mortality before the age of five years in different social groups as defined by maternal schooling.

**Methods:**

Data came from the MINIMat study (Maternal and Infant Nutrition Interventions, Matlab), a randomized trial of prenatal food supplementation (invitation early, about 9 weeks [E], or at usual time, about 20 weeks [U] of pregnancy) and 30 mg or 60 mg iron with 400 μgm folic acid, or multiple micronutrients (Fe30F, Fe60F, MMS) resulting in six randomization groups, EFe30F, UFe30F, EFe60F, UFe60F, EMMS, and UMMS (n = 4436). Included in analysis after omissions (fetal loss and out-migration) were 3625 women and 3659 live births of which 3591 had information on maternal schooling. The study site was rural Matlab, Bangladesh. The main stratifying variable was maternal schooling dichotomized as <6 years and ≥6 years. We used Cox proportional hazard model for survival analyses.

**Results:**

Overall, women having <6 years of schooling adhered more to food (81 vs. 69 packets, P=0.0001) but a little less to micronutrient (104 vs. 120 capsules, *P* = 0.0001) supplementation compared to women having more schooling, adjusted for maternal age (years), parity and body mass index (BMI, kg/m^2^) at week 8 pregnancy. Children of mothers with ≥6 years of schooling had lower under-five mortality, but the EMMS supplementation reduced the social difference in mortality risk (using standard program and schooling <6 years as reference; standard program and schooling ≥6 years HR 0.54, 95% CI 0.27-1.11; EMMS and schooling ≥6 years HR 0.28, 95% CI 0.12-0.70; EMMS and schooling <6 years HR 0.26, 95% CI 0.11-0.63), adjusted for maternal age (years), parity and body mass index (kg/m^2^) at week 8 pregnancy.

**Conclusions:**

The combination of an early invitation to prenatal food supplementation and multiple micronutrient supplementation lowered mortality in children before the age of five years and reduced the gap in child survival chances between social groups. The pattern of adherence to the supplementations was complex; women with less education adhered more to food supplementation while those with more education had higher adherence to micronutrients.

**Trial registration:**

ISRCTN16581394.

## Background

Interventions delivered by the health system are often more utilized by the well-off section of the population perpetuating injustice and social stratification in health [1]. This phenomenon reportedly occurs at least during the first few years after the start of an intervention, and remains true even for a relatively homogenously poor population [2]. The Commission for Social determinants of Health quests for solutions that reduce the gap in health induced by social inequity [3]. One way out is to target interventions to those most in need [4]. Other efforts may show variation in equity effects in different settings due to design as well as the historical and political trajectories of the local health system [5]. It is often assumed that the existing health system and its personnel will ensure that both poor and relatively rich will equally use interventions and will benefit from the interventions but in reality there can be variations. This is because the design of the intervention and the history of development of the health system in that particular country and the way the political trajectory has evolved may not allow equal distribution of intervention let alone equal effect. Often it is also assumed that economic growth will automatically lead to improvement in health and nutrition, which, unfortunately, is not the case. This has been demonstrated, for example, in case of India, where economic development was not accompanied by comparable improvement in child nutrition [6]. Thus, special interventions are needed for improving child nutrition particularly among the economically disadvantaged groups of the population. Social determinants of health may need special attention for improving early life nutrition using a pro-poor equity perspective [7]. It is therefore relevant to examine the effects of large interventions targeted to populations in low-income countries. Those targeted at pregnant women would be highly relevant because of potentially sustainable effects towards better health for the woman as well as for the offspring [8]. It is crucial to know whether such interventions also improve equity in health.

In this paper we analyzed the adherence to and the effect of prenatal food and micronutrient supplementations on child mortality in rural Bangladesh from an equity perspective. In order to investigate whether interventions differed in their impact on reducing social inequities we examined the differences in adherence to and the impact of prenatal food and micronutrient interventions on child mortality between mothers with low versus high level of education. We assumed maternal education would influence adherence to the intervention since it represents mother’s own socio-economic status (SES) and because of its influence on adherence to the intervention would also correctly differentiate the outcome of child mortality. We tested whether or not the results are consistent when analyzed by using household asset scores as another indicator since household asset represents family wealth, and interpreted the results considering that these indicators reflect different aspects of resources. We aimed at analyzing whether or not the interventions were able to achieve pro-poor (pro-disadvantaged) equity both in terms of utilization (adherence to supplements) and effect (reduction in mortality under the age of five years).

## Methods

### Design

Data came from the MINIMat study (Maternal and Infant Nutrition Interventions, Matlab), a trial where pregnant women were randomized to either an invitation to food supplementation early during pregnancy (E, at around 9 weeks), or at usual time during pregnancy (U, at around 20 weeks) and to one out of three types of micronutrient capsules, 30 mg iron and 400 μgm folic acid (Fe30F), or 60 mg iron and 400 μmg folic acid (Fe60F), or multiple micronutrients (MMS). Thus, randomization resulted in six arms, EFe30F, UFe30F, EFe60F, UFe60F, EMMS, and UMMS. The participants were enrolled from November 2001 to October 2003 and follow-up to five years of age was completed in June 2009. The main aim of the MINIMat study was to examine the effects of timing of prenatal food supplementation combined with different micronutrient supplementation alternatives on haemoglobin, birth weight and infant mortality, and secondary aims included maternal and child outcomes that included follow-up for a long time.

### Study site and participants

Total 4436 pregnant women were recruited into the study, resulting in 3591 deliveries. This number was associated with 3625 live births of which information on maternal school attendance was available for 3591; 13 more women had missing information on at least one of the following variables, maternal age (years), parity and BMI (kg/m^2^). Therefore, in this paper 3578 mother child pair were included in adherence to supplementation and mortality analysis adjusted for maternal age (years), parity and BMI (kg/m^2^) at week 8 of pregnancy. The study site was Matlab, a sub-district under Chandpur district, Bangladesh, where International Centre for Diarrhoeal Disease Research, Bangladesh (icddr,b) has been running a Health and Demographic Surveillance System (HDSS) for about four decades. The study was conducted within the part of Matlab where icddr,b is providing health services (about 110000 population). The participants were pregnant women enrolled in the MINIMat study. Details of the trial and effects on the main outcome have been reported earlier [9]. In the mortality analysis all live births, also twins, were included provided information on schooling was available.

### Procedures

All pregnant women of the study area were eligible for enrolment. If a woman reported to the community health research workers of icddr,b who visit her every month that her last menstrual period (LMP) was overdue, or that she was pregnant, she was offered a pregnancy test. A woman who tested positive was invited to join the study, and the date of her LMP was recorded. She was advised to visit the nearby icddr,b clinic as soon as possible, preferably at 8–10 weeks of gestation. At the clinic visit, if her pregnancy was confirmed by ultrasound and gestational age was <14 weeks she was individually randomized to one of the six trial arms. A computer generated register of study identity numbers with random assignment of food groups (“E” or “U”) and micronutrient groups (from 12 possible pill bottle number codes) was used for randomization. Randomization was done in blocks of 12 and independently for each of four icddr,b clinics. The micronutrient supplementation was double-blinded while the food supplementation was randomly allocated but not blinded. The pregnant women were expected to return to the clinic on three more occasions (14, 19 and 30 weeks of gestation), and were visited monthly at home by study interviewers. This study was approved by the ethical review committee of icddr,b. Informed written consent was obtained from all participants.

### Interventions

The ongoing, government-supported national programme provided an energy-protein supplement (about 600 kcal/d) to all pregnant women irrespective of body mass index (BMI-kg/m^2^). The food supplement was made available through community nutrition centres (CNC) 6 d/wk. This was different from the national programme policy where only pregnant women with BMI <18.5 kg/m^2^ were supposed to receive the supplements. In the MINIMat trial pregnant women were individually randomized to be invited to the feeding programme immediately after pregnancy detection, early assignment, or at the time of their choosing, usual assignment. Nutrition centres (one for each 1200 population) managed the food supplementation. In general, till the end of 2^nd^ trimester women visited CNCs and consumed food supplement at that place. The food supplement sessions were organized by BRAC (a non-governmental organization) as the implementing organization. Only during the 3^rd^ trimester the food was delivered at pregnant women’s homes by the project staff.

Women were also individually randomized to one of the three types of micronutrient supplements that were distributed at the icddr,b sub-centres: (a) 30 mg iron and 400 μg of folic acid (Fe30F); (b) 60 mg of iron and 400 μg of folic acid, (Fe60F); and (c) MMS which contained 15 micronutrients as recommended by UNICEF/WHO/UNU for trial purposes: 30 mg iron, 400 μg folic acid, 800 μg RE vitamin A, 200 IU vitamin D, 10 mg vitamin E, 70 mg vitamin C, 1.4 mg vitamin B1, 1.4 mg vitamin B2, 18 mg niacin, 1.9 mg vitamin B6, 2.6 μg vitamin B12, 15 mg zinc, 2 mg copper, 65 μg selenium and 150 μg iodine [10].

### Monitoring of adherence

At every monthly home visit, the interviewers asked a series of questions to assess adherence to food supplements in the previous 30 days. The specific question asked on a monthly basis was “For the last 30 days, how many packages have you eaten?”. Data from these interviews were summed up (total 24 weeks, week 10 to 34) and the adherence to food packets over pregnancy was derived. Food supplement data upto week 34 were included in order to avoid influence on adherence data by preterm delivery.

Daily micronutrient supplements were offered at the 14 weeks clinic visit. The three types of micronutrient supplements looked identical, and were distributed in special pill bottles (eDEM®, Aprex, Fremont, California, US). Each bottle contained 35 capsules and replacement bottles were provided at home during monthly visits by interviewers. The eDEM® device was used for monitoring of adherence to the micronutrient supplementation. The pill bottle cap was equipped with a counting device and a microprocessor. Each time the pill-bottle was opened and closed, the time and date were recorded. The information in the caps was downloaded into a computer from bottles collected from the enrolled women. Adherence to micronutrient capsules was derived from the total number of openings of pill bottles from week 14 of pregnancy till childbirth.

### Notifications of births and measurements of socioeconomic status

A birth notification system was established to ensure that study staff was made aware of births as soon as they occurred. At start of labour pain family members informed the CHRW, who usually lived in the same village. The CHRW alerted trained paramedics, who measured anthropometry and recorded birth outcome [9]. Health personnel measured birth anthropometry of children born at health facilities. Maternal education was assessed as completed years of schooling, and dichotomized to primary education, i.e. <6 years, and more than primary education, ≥6 years of schooling. Measurement of household assets included possession of television, radio, domestic animals, chairs, tables, beds, and bicycles, or rickshaw. From these set of variables an asset score was developed by using principal component analysis [11]. We dichotomized the asset score in our analysis.

### Outcomes

The primary outcomes of the MINIMat trial were maternal haemoglobin level at 30 weeks of gestation, birth weight and infant mortality. The effects of the interventions on these outcomes have been reported [9]. In this paper adherence to the interventions and the effect on mortality under the age of five years are analysed in the two subgroups defined by maternal education level in order to analyse whether adherence to the intervention and effects were pro equity.

Information on infant and child mortality was collected at follow-up visits at 7–12 day postpartum, and at monthly visits during infancy. Community health research workers also collected information on child survival on a monthly basis as part of the routine surveillance system in Matlab. These workers visited households and collected information on vital events like births, deaths (also mortality of infants and children), and migration.

### Variables

The main outcome in this analysis was death of a live birth before the age of five years. The main exposure variables were the food and micronutrient interventions and the main stratifying variable was maternal years of schooling, dichotomized as <6 years (median value) and ≥6 years, which also corresponds to primary and above primary education, respectively. We used the median as cut off point when dichotomizing the stratifying variable, since there was not sufficient power to divide into tertiles. To test whether the results were consistent if a different SES variable was used instead of maternal education, we used dichotomized value of asset scores that represents family wealth. Presence of daily wager in the family, and reported income expenditure status, maternal age, parity, body mass index (BMI, kg/m^2^) at entry into the intervention, and haemoglobin in early pregnancy were used as intermediate variables to examine if women having lower and higher schooling differed by the levels of these variables.

### Statistical analyses

**Figure 1:**
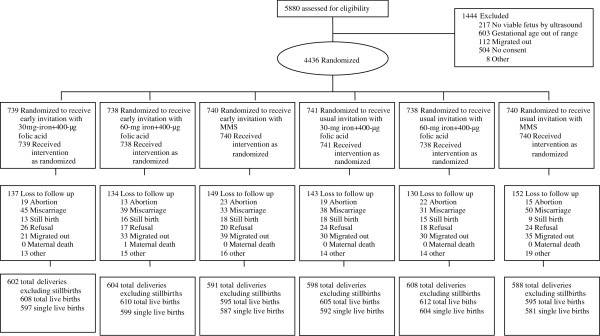
Study flow of women and infants.

All mortality analyses were done based on intent-to-treat using dichotomized maternal schooling as the stratifying variable. Analyses were done for women having live births (Figure 1). Baseline characteristics between maternal schooling groups, i.e., <6 and ≥6 years of schooling, were evaluated by *student’s t test* for continuous variables and *chi square* test for categorical variables since these were intermediate variables with potentials to influence our results even though our results are based on a randomized trial. Adherence to food and micronutrient supplementation for maternal schooling groups were compared by *student’s t test* and also by univariate analysis of variance adjusted for maternal age (years), parity and BMI (kg/m^2^) at week 8 pregnancy. Mean adherence to food and micronutrient supplements by a combination of maternal years of schooling and randomization groups, interactions between food groups and micronutrient groups, between food groups and maternal education groups and between micronutrient groups and maternal education groups were analyzed by univariate analysis of variance. Except for those testing interactions, all analysis of adherence to the interventions were adjusted for maternal age (years), parity and BMI (kg/m^2^) at week 8 pregnancy. We tested the association between household asset scores and maternal schooling by linear regression. Mortality before the age of five years was analysed across the food and micronutrient supplementation groups and repeated after stratification for groups defined by level of maternal education. The effect of the interventions on mortality was examined by Cox proportional hazard model by calculating hazard ratios (HRs) and 95% confidence intervals (CIs) comparing with the rate in standard intervention, UFe60F (reference category) and other trial arms and published in our previous report [9]. In this paper we analyzed child mortality after stratification for maternal schooling groups, using UFe60F and lower schooling as reference, and taking maternal age (years), parity and BMI (kg/m^2^) at week 8 pregnancy as co-variates. We tested the analysis by replacing maternal schooling groups by households’ asset scores dichotomized based on median value. Statistical significance was set at *P* < 0.05. Analyses were done using PASW statistics version 18.0 (IBM corporation).

### Role of the funding sources

None of the donors played any role in analysis or publication of the results.

## Results

Baseline characteristics were equally distributed between randomization groups (Table 1). As expected, compared to women having ≥6 years of schooling, women having <6 years of schooling were significantly older (mean age 24.2 years, SD 5.1, vs. 28.2 years, SD 6.0, respectively, *P* < 0.001), had higher parity (mean 1.5, SD 7.9, vs. 2.0, SD 2.9, respectively, *P <* 0.01), and had lower BMI at week 8 of pregnancy (mean 20.5, SD 2.8, vs. 19.9, SD 2.6, respectively, *P* < 0.001). The women having less schooling were in general from families having lower mean asset scores (−1.23 vs. 1.36, *P* < 0.001), more frequent deficits in a scale for income expenditure status (*P* < 0.001), and more frequent presence of daily wager in the family (*P* < 0.001).

**Table 1:** Baseline characteristics by maternal schooling groups

	**<6 years**	**≥6 years**
Age (years)	28.13 (5.89) [1884]	24.30 (5.16) [1703]
BMI at week 8 pregnancy (kg/m^2^)	19.92 (2.58) [1890]	20.41 ( 2.69) [1706]
Parity		
0	322/1176 (27.4)	854/1176 (72.6)
1	511/1043 (49.0)	532/1043 (51.0)
≥2	1051/1369 (76.8)	318/71369 (23.2)
Hemoglobin at baseline (g/dL)	11.57 (1.26) [1726]	11.77 ( 1.29) [1578]
Father’s schooling (years)		
<6	1448/1889 (76.7)	441/1889 (23.3)
≥6	423/1686 (25.1)	1263/1686 (74.9)
Household asset score (median value)		
<0.45	1641/2239 (73.3)	598/2239 (26.7)
≥0.45	483/2233 (21.6)	1750/2233 (78.4)

*Abbreviation*: *BMI* body mass index (kg/m^2^), calculated as weight in kilograms divided by height in meters squared. Data are mean (SD) [n] analyzed by *student’s t test*, or (%) analyzed by *Chi*^*2*^*test*. Information on schooling and asset scores is missing on 34 out of 3625 women included in child mortality analysis. For all comparisons between schooling groups, *P* < 0.0001.

### Adherence to food and micronutrient supplements

Overall, women randomised to EMMS adhered to more food supplement than women randomised to the standard intervention (UFe60F), 94 vs. 60 packets, *P* < 0.001. In general, women having <6 years of schooling adhered more to food (83 vs. 68 packets, *P* < 0.001) but less to micronutrient supplementation (105 vs. 119 capsules, *P* < 0.001) than women having more schooling. This was true when adjusted for maternal age (year), parity and BMI (kg/m^2^) at week 8 pregnancy both for food (81 vs. 69 packets, *P* < 0.0001) and micronutrient interventions (104 vs. 120 capsules, *P* < 0.0001).

When analysed by the intervention groups, stratified for maternal years of schooling groups this pattern remained; women having less schooling reportedly consumed more food supplements than women having more schooling (Table 2). Also, women having less schooling who were randomised to EMMS consumed more food supplements than women having the same level of education and randomised to the standard program, UFe60F, 102 vs. 65 packets, *P* < 0.001 (Table 2).

**Table 2:** Adherence to food and micronutrient supplementation in the randomised treatment groups and after stratification into maternal schooling groups

	**Early invitation to prenatal food supplementation**			**Usual invitation to prenatal food supplementation**		
	**30 mg iron + 400 μg folic acid**	**60 mg iron + 400 μg folic acid**	**Multiple micro-nutrients**	**30 mg iron + 400 μg folic acid**	**60 mg iron + 400 μg folic acid**	**Multiple micro-nutrients**
Food packages^a^	91 (1.55)	88 (1.54)	94 (1.55)	61 (1.54)	60 (1.53)	61 (1.57)
Mother’s schooling <6 years	97 (2.14)	97 (2.24)	102 (2.17)	65 (2.14)	65 (2.14)	67 (2.23)
Mother’s schooling ≥6 years	83 (2.23)	79 (2.22)	88 (2.32)	58 (2.32)	55 (2.30)	54 (2.27)
Micronutrient capsules^b^	109 (2.27)	113 (2.25)	107 (2.24)	117 (2.25)	113 (2.21)	110 (2.29)
Mother’s schooling <6 years	100 (3.02)	104 (3.16)	97 (3.03)	110 (3.03)	108 (2.99)	104 (3.15)
Mother’s schooling ≥6 years	121 (3.45)	121 (3.19)	118 (3.34)	126 (3.37)	118 (3.27)	117 (3.31)

Data are mean (SE). Analyses are adjusted for maternal age (years), parity, and body mass index (BMI, kg/m2) at week 8 pregnancy; n = 3578 since out of 3591 women included in child mortality analysis 3578 had information on adjusting factors. Multiple micronutrients include 15 micronutrients, including 30 mg iron and 400 μg folic acid. ^a^Assessed from enrolment to week 34 examination; mean difference between maternal schooling groups (<6 years vs. ≥6 years) 14 packages, average 81 vs. average 69 packages, (*P* = 0.0001). Stratified analysis by maternal schooling groups, mean difference between <6 years UFe60F and <6 years EMMS, significant (*P* = 0.0001). ^b^Assessed from supplement distribution at week 14 examination to childbirth; mean difference between maternal schooling groups (<6 years vs. ≥6 years) 14 capsules, average 104 vs. 120 capsules, (*P =* 0.0001). Stratified analysis by maternal schooling groups, mean difference between <6 years UFe60F and <6 years EMMS, not significant (*P*=0.83).

In contrast, women having more schooling consumed more micronutrient supplements than women having less schooling, but the differences were statistically significant only for EFe30F, *P <* 0.01 and EMMS, *P <* 0.01 (Table 2).

Adherence to food supplement varied by micronutrient supplementation groups with marginal level of significance, 76, 74 and 78 packages, (*P* = 0.05), as expected, varied by food supplementation groups (*P* < 0.01) and the interaction between food and micronutrient groups was marginally significant (*P* = 0.06). There was no significant interaction between food invitation groups and schooling groups (*P* = 0.29). Adherence to micronutrient supplement varied by micronutrient supplementation groups (*P* = 0.03), food supplementation groups (*P* = 0.02) but there was no interaction between food and micronutrient groups (*P* = 0.69). Also, there was no significant interaction between micronutrient supplementation groups and schooling groups (*P* = 0.63).

### Mortality under the age of five years

The mortality rate under the age of five years was 44.4 per 1000 live births (161 deaths, 3625 live births). Information on education level of the mother was missing for 34/3625 live births. For the remaining 3591 live births the under-five mortality in the group with maternal schooling <6 years was 60.3 (116 out of 1923) and for those where the mother had schooling ≥6 years it was 33.6 (56 out of 1668) per 1000 live births (*P* < 0.001).

### Effects of the interventions on mortality in different maternal schooling strata

Women having less schooling who were randomized to EMMS had a larger reduction in the risk of under-five child death than women having the same level of education and randomized to UFe60F. Within that social group the EMMS intervention moved the survival probabilities of those children to the level of children of women having above primary schooling (Table 3).

**Table 3:** **Risk of mortality before the age of five years by randomised food and micronutrient supplementation groups and after stratification into maternal schooling groups**^**1**^

	**Early invitation to prenatal food supplementation**			**Usual invitation to prenatal food supplementation**		
	**30 mg iron + 400 μg folic acid**	**60 mg iron + 400 μg folic acid**	**Multiple micro-nutrients**	**30 mg iron + 400 μg folic acid**	**60 mg iron + 400 μg folic acid**	**Multiple micro-nutrients**
	Hazard Ratio (95% CI)					
All (n = 3625)	0.92 (0.57-1.5)	0.92 (0.57-1.5)	0.34 (0.18-0.66)	0.66 (0.39-1.1)	1.0 (reference)	1.1 (0.72-1.8)
Strata (n = 3591)^a^						
Mother’s schooling <6 years	0.86 (0.47-1.56)	0.79 (0.42-1.47)	0.26 (0.11-0.63)	0.71 (0.38-1.34)	1.0 (reference)	1.32 (0.76-2.30)
Mother’s schooling ≥6 years	0.56 (0.27-1.13)	0.65 (0.33-1.25)	0.28 (0.12-0.70)	0.28 (0.11-0.70)	0.54 (0.27-1.11)	0.45 (0.21-0.96)

^1^Adjusted for maternal age (years), parity and body mass index (kg/m^2^) at week 8 pregnancy.

^a^Information on schooling and asset scores is missing on 34/3625 women of them 3578 had information on maternal age (years), parity and body mass index (kg/m^2^) at week 8 pregnancy. Multiple micronutrients include 15 micronutrients, including 30 mg iron and 400 μg folic acid.

In order to test the consistency of the results so far the analysis was repeated by replacing maternal education level by household asset scores. Women allocated to the standard program (UFe60F) and who had an asset score below median value, <0.45, were used as the reference. Women allocated to EMMS and also living in household with the lower asset score had hazards of child death before the age of five years (HR 0.31, 95% CI 0.13-0.73) that was lower than those of a higher asset score and standard supplementation (HR 0.58, 95% CI 0.30-1.31). The hazard of underfive death when women had been allocated to EMMS and had a household asset score ≥0.45 was the lowest (HR 0.22, 95% CI 0.08-0.57) but was not significantly lower than those having the same level of asset score and randomized to standard supplementation nor from those having lower asset score and randomized to EMMS. Thus, the results using maternal schooling as a marker of socio-economic level is confirmed when asset score has been used as the alternative marker for representing SES.

Asset scores dichotomized values were positively associated with maternal schooling groups, b/SE of b, 0.517/0.013, *P* = 0.0001, indicating with increasing asset scores maternal education also increases and education is a reflection of SES represented by asset scores.

## Discussion

In the MINIMat trial of prenatal food and micronutrient supplementation, we observed higher adherence to food supplementation but lower adherence to micronutrient supplements among women having less than primary education. The combination of an early invitation to prenatal food supplementation and multiple micronutrients lowered mortality under the age of five years and reduced the child survival gap between maternal education groups.

### Validity of data

Results were based on intent-to-treat analyses that offer unbiased estimates of the effect. The baseline characteristics between trial arms were comparable indicating efficient randomization. For social stratification we used maternal years of schooling. The results remained comparable when instead stratified for asset score groups. Food supplementation data came from repeated questions based on 30 days recall. Food supplements were – especially in the earlier parts of pregnancy – consumed at the local community nutrition centres. Most of the differences in adherence to food supplement occurred during the first few weeks, until about week 22. Adherence was easier to measure during the first two trimesters of pregnancy and most of the adherence to food supplement data came from this part, and therefore differential reporting by women having lower and higher schooling, if happened at all, most likely would not influence our results.

Data on micronutrients came from pill bottles having microchips attached to the caps that recorded each opening associated with taking one capsule. Mothers were unaware of this device.

### Adherence

Adherence to prenatal food supplements was higher among women who had less schooling. This confirms our finding in a previous study conducted in another area of Bangladesh [12]. Women with less schooling probably had more need of supplementary food due to frequent problem of food insecurity. The reported consumption of food supplement among women having less schooling who were randomized to EMMS was 102 packets, providing about 62,000 extra kcal for the measured time period. This may be compared to the average calorie intake from food supplement in our previous study, 116 days of supplementation providing about 70,000 extra kcal over pregnancy [12], and the mean supplementation intake in a prenatal food supplementation trial in the Gambia providing 72,000 kcal [13,14]. Women having less schooling who were randomized to EMMS consumed 37 more food packets equivalent to 22,000 kcal extra compared to women having the same level of education who were randomized to the standard intervention. However, the positive effects on child survival may also be related to different responses to the early timing of food supplements among women of the two educational levels that also are linked to characteristics in level of malnutrition and other aspects of health [9].

Women having less education had lower BMI at entry, low haemoglobin at baseline, older and had higher parity and also adhered to more food supplement. Although we do not have data on haemoglobin, these characteristics were also related to higher intake of food supplement in our previous study [12]. These indicate the higher adherence to food supplement was also driven by the above factors that entailed more needs for food supplement.

Our results provide empirical evidence of reduction in offspring mortality when such evidence from prenatal food supplementation is scarce and that from micronutrient supplementation is equivocal. Overall 66% reduction in child mortality [9] is greater than 46% reduction in neonatal mortality found in a prenatal food supplementation study in the Gambia [13], and 22% reductions of child mortality from MMS supplements in a trial in Indonesia [15]. This is also greater compared to 34% reduction in neonatal mortality in the home care arm than the comparison arm in an integrated package of newborn care study in Bangladesh [16] and compared to 62% reductions with the same type of care in India [17,18].

Higher adherence to food supplement by less educated women in our study may be due to more frequent and severe food insecurity among them. Due to the overwhelming need for food these women may have consumed more food packets compared to their more educated counterpart who were relatively secured in terms of availability of food. A lower adherence to micronutrient supplements was observed among women with less schooling. This is consistent with the findings from a previous iron supplementation study in Bangladesh [19] and from a study among female garment employees in Cambodia [20]. A possible explanation may be that women having more schooling regarded capsules as indicative of modern care, while women with less schooling did not share that view and were also not aware of the added advantages of the tablets. However, a study in Vietnam reported concern for newborn health as a significant reason to comply with iron folic acid supplements [21]. Because of similar reasons probably women in our study adhered with some micronutrient capsules since concern for newborn health is a unique phenomenon as women perceive [22], and all women were equally worried for that but the differences between less and more educated women were not significant.

### Effects and differences in effects in relation to maternal years of schooling

A recent meta-analysis of MMS trials concluded no effect of MMS on neonatal mortality [23]. When compared to micronutrient supplementation trials in developing countries [15,24-26] the overall effect of the combined early food supplementation and multiple micronutrients on survival was large.

There is evidence of higher uptake in maternal and child health interventions among the advantaged segment of the community [27-30] who in general have better access to health services. Higher uptake of intervention and effects in disadvantaged group has also been reported. In a study in Colombia increased uptake of health care occurred among disadvantaged children [31]. In Bangladesh decreased hazard of infant deaths was observed for poor women participating in development programs [32].

As has been done for integrated management of childhood illnesses in Peru [4,33], prenatal food supplementations in poorer districts in Bangladesh may ensure pro-disadvantaged equity in child survival. Our results contradict the “inverse equity hypothesis” [2], and show that increasing equity in survival is possible without initial widening of gaps [3]. This was possibly due to the responsiveness of the women having less education who were at a higher risk of child death and were responsive to the intervention [34].

### Appropriateness of education as a stratifying variable

Although socio-economic indicators are widely used in medical research, which SES marker to use is a matter of debate [35,36] especially for assessing inequalities in health [37]. In our analysis maternal years of schooling clearly identified the disadvantaged group. The results are confirmed when another SES variable, asset score, has been used as an alternative but greater effect estimate (reduction in hazard of child death), HR 0.26 vs. HR 0.31, was achieved by using our main stratifying variable, maternal schooling. Level of schooling relates to the individual’s position in family and society, while asset scores characterise the household.

### Strengths and limitations of the study

The main strength of the study is that in a developing country setting where maternal undernutrition, food insecurity, and child mortality are prevalent, MINIMat was a randomised trial with timing of invitation to prenatal food supplementation combined with one of the three types of micronutrients including one already available at the field. This design enabled an unbiased examination of the effects of EMMS against the usual practice, UFe60F. Adherence to micronutrients was assessed by a high-validity method. Adherence to food supplementation was not directly observed but assessed by repeated recalls up to week 34, while supplementation continued up to childbirth, which is a methodological limitation.

## Conclusions

The combination of an early invitation to prenatal food supplementation and multiple micronutrient supplementation was pro-equity; it lowered mortality in children before the age of five years and reduced the gap in child survival chances between social groups. The pattern of adherence to the supplementations was more complex; women with less education adhered more to food supplementation while those with more education had higher adherence to micronutrients.

## Competing interests

The authors declare that they have no competing interests.

## Authors’ contributions

RS was responsible for measurement of food supplement intake, supervised field work, did initial analysis and wrote the first draft of the manuscript. PKS was responsible for the Health and Demographic Surveillance System, icddr,b, verification of mortality data, contributed in analysis and writing up. RTN was responsible for the development of socio-economic variables, supervised field work, and contributed in analysis and writing up. LL contributed in analysis, interpretation and writing up. LAP conceived the study, was responsible for data ownership and measurement of food and micronutrient supplement intake, contributed in analysis and writing up. At the conception of the study, all authors participated in the discussion and contributed in different capacities. All authors have read and approved the final version.

## Pre-publication history

The pre-publication history for this paper can be accessed here:

http://www.biomedcentral.com/1471-2458/14/5/prepub
